# The Medical Defence Union and the London and Counties Medical Protection Society

**Published:** 1893-12-16

**Authors:** W. Bruce Clarke, G. A. Heron, Hugh Woods

**Affiliations:** Harley Street


### Editor's Letter-Box.

[Our correspondents are reminded that proxility is a great bar to publication, and that brevity of style and conciseness of statement greatly facilitate early insertion.]

### THE MEDICAL DEFENCE UNION AND THE LONDON AND COUNTIES MEDICAL PROTECTION SOCIETY.

Sir,—In The Hospital of the 9th inst. there appears a letter which is signed by Drs. Leslie Phillips and Bateman, Secretaries to the Medical Defence Union. There are in the letter certain statements which, in our opinion, ought to be dealt with at once in order that, in discussing recent occurrences in these two Societies, facts may not be lost sight of in the cloud of words which threatens to obscure them. We now repeat in substance Dr. Woods' words as they are given in the letter of Drs. Phillips and Bateman, and we say that the conference which was held with a view to the amalgamation of these two Societies was not terminated by the refusal of the Sub-Committee of the London and Counties Medical Protection Society to accept the revision of the names of their representatives by the Medical Defence Union. It is true that Dr. Heron said in effect, and Mr. Bruce Clarke agreed with him, that such a proposal as this demand for scrutiny of the list of representatives was, in his opinion, so unreasonable that neither our Council, nor any other body of men who respected themselves, would, in the circumstances, submit to it. However, after some conversation had passed touching this matter, Dr. Heron, in order to minimise its unreasonableness, proposed that the Medical Defence Union should submit its "list of councillors for the proposed joint society to the same measure of criticism to which the Medical Defence Union intends to submit the list of councillors to be proposed by the Medical Protection Society."

This counter-proposal was formally put forward on November 7th, at the last meeting of the amalgamation committees of the two Societies, was written out by Dr. Leslie Phillips, Secretary to the Medical Defence Union, and Mr. Victor Horsley and Dr. Leslie Phillips, in answer to definite questions put by us, stated that the counter-proposal would be laid before their Council, and that in about ten days' time our Society should have their answer. We certainly believed and said that if the Medical Defence Union did not accept our counter-proposal, we did not think that our Council would grant that Society a right of scrutiny which the Defence Union refused to us.

To say all this was was an "after-thought and had no meaning" is to make a statement which, unlike the works of fiction of some years ago, is not even founded on fact. To this day we do not know whether the Sub-Committee of the Medical Defence Union did or did not lay our counter-proposal before their Council. We had Mr. Horsley's promise that this would be done, and we believe, therefore, that it was done. Why, then, have the terms of our counter-proposal and of the answer of the Council of the Medical Defence Union been so carefully excluded from the so-called *résumé* of the proceedings of the two Sub-Committees which has been published by the Medical Defence Union? Mr. Horsley knows that these important facts have been omitted from the *résumé*, to the correctness of which he pledged himself when he sent it to you for publication.

In our letter of December 9th we have stated the whole of the facts concerning the matter of what in the *résumé* is called the "fact that the London and Counties Medical Protection Society contemplated winding up." We have there shown that this statement is absolutely without even a shadow of foundation.

As to the rest of the letter of the two Secretaries of the Medical Defence Union, we do not think it is necessary that we should deal with it. We prefer to allow your readers, who are interested in the subject, to judge of the tone of the whole letter by what we have shown is true of those parts of it which are material to the real points at issue.

W. Bruce Clarke.

G. A. Heron.

Hugh Woods.

Harley Street, December 12th, 1893.

